# Purification and characterization of DR_2577 (SlpA) a major S-layer protein from *Deinococcus radiodurans*

**DOI:** 10.3389/fmicb.2015.00414

**Published:** 2015-06-03

**Authors:** Domenica Farci, Matthew W. Bowler, Francesca Esposito, Sean McSweeney, Enzo Tramontano, Dario Piano

**Affiliations:** ^1^Laboratory of Plant Physiology and Photobiology, Department of Life and Environmental Sciences, University of CagliariCagliari, Italy; ^2^Laboratory of Synchrotron Crystallography, Department of Structural Biology, European Molecular Biology LaboratoryGrenoble, France; ^3^Unit for Virus Host Cell Interactions, Laboratory of Structural Biology of RNA-Protein Complexes in Gene Expression and Host-Pathogen Interactions, University Grenoble Alpes-EMBL-Centre National de la Recherche ScientifiqueGrenoble, France; ^4^Laboratory of Molecular Virology, Department of Life and Environmental Sciences, University of Cagliari, Cittadella Universitaria di MonserratoCagliari, Italy; ^5^Department of Photon Sciences, Brookhaven National LaboratoryUpton, NY, USA; ^6^Laboratory of Structural Biology, International Institute of Molecular and Cell BiologyWarsaw, Poland

**Keywords:** S-layer, hexagonally packed intermediate, SlpA, DR_2577, *Deinococcus radiodurans*

## Abstract

The protein DR_2577 is a major Surface layer component of the radio-resistant bacterium *Deinococcus radiodurans*. In the present study DR_2577 has been purified and its oligomeric profile characterized by means of size exclusion chromatography and gel electrophoresis. DR_2577 was found to be organized into three hierarchical orders characterized by monomers, stable dimers formed by the occurrence of disulfide bonds, and hexamers resulting from a combination of dimers. The structural implications of these findings are discussed providing new elements for a more integrated model of this S-layer.

## Introduction

Surface layers (S-layers) are paracrystalline two-dimensional arrays of proteins associated to the external side of the cell wall covering the surface of many bacterial species (Sleytr, 1978; Sleytr et al., 1993; Bahl et al., 1997). Irrespective of cell wall architecture, S-layers are equally spread among bacteria (Sára and Sleytr, 2000).

Being functionalized structures, S-layers evolved with different ecological aims spanning from passive functions, such as cell rigidity and cell shape, to active functions such as cell adhesion and cell protection (Beveridge et al., 1997; Sleytr and Sára, 1997; Fagan and Fairweather, 2014). In *Deinococcus radiodurans* the S-layer has an unknown function but the involvement in providing or contributing to its extraordinary ability to resist high doses of ionizing radiation and UV radiation cannot be excluded. The S-layer of *D. radiodurans* is characterized by a regular repetition of pores believed to be composed of only one protein called the Hexagonally Packed Intermediate (HPI) coded by the gene DR_2508 (Baumeister et al., 1992, 1996). However, a second component, the protein DR_2577, also known as SlpA, emerged to be essential in the S-layer organization and integrity (Rothfuss et al., 2006). Being naturally over expressed, DR_2577 is present in the S-layer at high levels representing a primary component of this structure (Farci et al., 2014). Such a role is also consistent with the homology that DR_2577 shares with the SlpA protein from *Thermus thermophilus* in which this protein is the main component in the S-layer architecture (Faraldo et al., 1991, 1992). In agreement with these observations, a revisited analysis regarding the relationship between structure and protein composition of this S-layer, must consider the primary role of the protein DR_2577 which coats, in association with HPI and several other proteins, the external surface of *D. radiodurans* building the resulting S-layer structure in the well characterized form of a regular paracrystalline two-dimensional repetition of proteins (Baumeister et al., 1992, 1996). In order to gain more insights into the DR_2577, we have developed a fast procedure for its isolation, confirming its major contribution in the organization of this S-layer. From the characterization of the protein it has emerged that it forms monomers and stable dimers due to the presence of disulfide bonds. Moreover, a further step of purification by size exclusion chromatography led to the conclusion that the main form of DR_2577 is represented by a higher oligomeric state with respect to the dimer observed by SDS-PAGEs. Furthermore, BN-PAGEs shows that DR_2577 occurs in the form of ~760 kDa complexes ascribed to be hexamers of DR_2577 resulting from a trimer of dimers. The implications of this finding are discussed on the basis of the existing model of the *D. radiodurans* S-layer and the essential role of DR_2577 in its integrity.

## Materials and methods

### Bacterial strain and growth conditions

*D. radiodurans* strain R1 (ATCC 13939) was grown in tryptone/glucose/yeast extract broth (TGY) (Murray, 1992) for 24 h at 30°C, with shaking at 250 rpm. Cells were harvested by centrifugation of 1 l cultures at 5000 × g for 10 min at 4°C and resuspended in 50 mM Na Phosphate pH 7.8 (Buffer A).

### DR_2577 enriched membranes preparation

Whole cell membrane fractions were purified at 4°C according to Farci et al. (2014). After centrifugation the cells were resuspended in Buffer A, treated with DNase and disrupted using a French Pressure Cell. Unlysed cells were removed by low speed centrifugation (4°C, 2 × 2000 × g for 10 min). The final supernatant was centrifuged again (4°C, 48,000 × g for 10 min) and the pink pellet resuspended in 10 ml of Buffer A. A second step of lysis was performed by using a French Pressure Cell followed by centrifugation and resuspension (4°C, 48,000 × g for 10 min). To remove surface polysaccharides the membrane suspension was incubated under agitation (800 rpm) with 100 μg/ml lysozyme for 8 h at 30°C. The membrane suspension was then centrifuged (4°C, 48,000 × g for 10 min) in order to obtain the protein DR_2577 in solution.

### Size exclusion chromatography

The protein sample obtained after the solubilization step was loaded on to a 20 ml size exclusion chromatography column (Superose 6 10/300GL, GE Healthcare) previously equilibrated in Buffer B [50 mM Na Phosphate pH 7.4, 0.06% (w/v) β-DDM] at a flow rate of 0.3 mL/min. The molecular weight of the DR_2577 complex resolved by the size exclusion chromatography was estimated by plotting the elution volume vs. the logarithm of the molecular weight of the standard proteins (Gel Filtration Standard, Biorad) using a polynomial curve fit (second-order polynomial best-fit).

### Polyacrylamide gel electrophoresis (PAGE)

For denaturing Sodium Dodecyl Sulphate-Polyacrylamide Gel Electrophoresis (SDS-PAGE), 5 or 10% (w/v) separating polyacrylamide/urea gels with 4% (w/v) stacking gels were used (Schägger and Von Jagow, 1987). Monomeric samples were resolved by denaturing with Rotiload (Roth) and boiling for 10' or alternatively by treating them with 6 mM tris-2-carboxy-ethylphosphine at room temperature before loading. Dimeric samples were resolved by denaturing with Rotiload (Roth) but avoiding to boil or to treat them with tris-2-carboxy-ethylphosphine. After the electrophoretic separation the gels were stained with Coomassie Brilliant Blue G250. Blue Native-Polyacrylamide Gel Electrophoresis (BN-PAGE) was carried out using 3–12% (w/v) continuous gradient gels, according to Schägger and Von Jagow (1991). Pink envelopes were mixed with 0.25 volumes of Coomassie Blue Solution 5%, (v/v) Serva Blue G, 750 mM aminocaproic acid and 35% (w/v) sucrose. Electrophoresis was carried out at 205 V for 5 h at 4°C. The molecular weight of the native DR_2577 complex resolved by the BN-PAGE or of the denatured DR_2577 samples resolved by SDS-PAGE was estimated by plotting the retardation factor values (Rf, length of the band migration/length of the dye front) vs. the logarithm of the molecular weight of the molecular marker (NativeMARK, Invitrogen for the BN-PAGE and the Prestained Standard high range, Biorad for the SDS-PAGES) using a polynomial curve fit (second-order polynomial best-fit), according to manufacturer's instructions.

## Results

### Isolation of DR_2577 from the cell wall fragments

**Figure 1:**
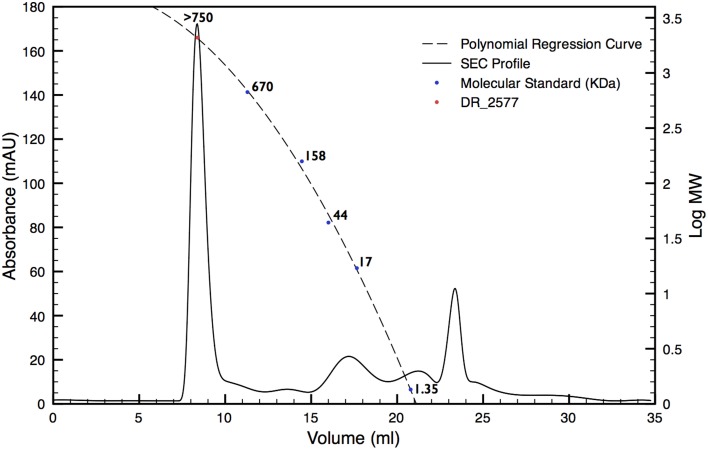
**SEC profile for DR_2577 enriched samples (dashed line)**. The elution profile shows a main peak eluted at small retention volumes suggesting the presence of a high molecular weight complex. A polynomial regression curve is shown (dotted line) calculated on the basis of the retention volumes of the molecular standard (blue dots). The red dot on the polynomial regression curve shows the calculated mass for DR_2577.

**Figure 2:**
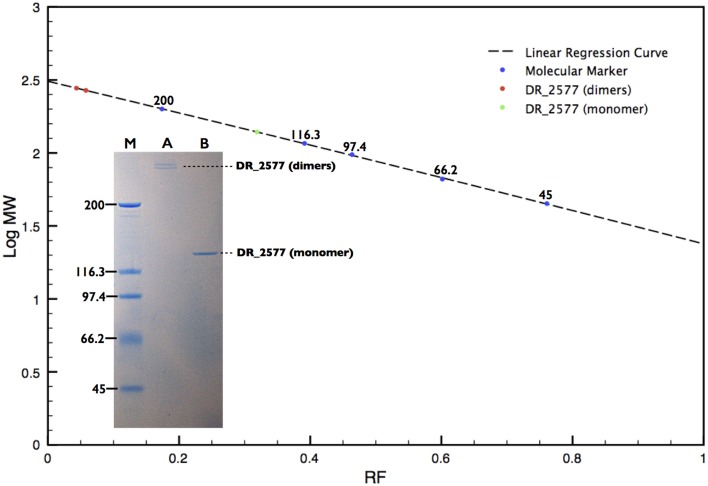
**SDS-PAGE analysis of the DR_2577 sample eluted from the main SEC peak**. A linear regression curve (dotted line) calculated on the base of the retardation factors of the molecular standard (blue dots) is shown. The two red dots and the green dot on the polynomial regression curve show the calculated mass found for the DR_2577 dimers and monomers, respectively. The insert shows that standard procedures of sample denaturation lead to the separation of two near bands above 200 kDa (A); samples treated under harsher conditions (see text) bring to a single band of 120 kDa (B); the lane M indicates the standard with the corresponding molecular weights.

In order to characterize the protein DR_2577, the whole cell membrane fraction was purified and its quality checked by electron microscopy as described in Farci et al. (2014). The intact cell membrane fragments obtained were subsequently subjected to several cycles of disruption and centrifugation. By this procedure, starting from a cell membrane fraction already enriched in DR_2577 (Farci et al., 2014) (Supplementary Figure 1), it was possible to obtain a selective delivery of DR_2577 from the bacterial cell wall fragments to the soluble fraction resulting in an almost pure sample (Supplementary Figure 1). A subsequent step of Size Exclusion Chromatography (SEC, Figure 1) was able to increase further the level of purity as confirmed by SDS-PAGE (Figure 2). After purification, the identity of the isolated DR_2577 was confirmed by Mass spectrometry (data not shown).

### DR_2577 dimers are exceptionally stable

SDS-PAGEs on the protein samples, treated according to the standard procedures of denaturation, showed two very near bands that migrated with a double apparent molecular weight with respect to the expected DR_2577 mass of 124 kDa, indicating that the protein may occur as stable dimers (Figure 2, Table 1). Complete dissociation to monomers was reached only under harsh conditions of denaturation such as long boiling or strong treatment with tris-2-carboxy-ethylphosphine. In these conditions the protein samples when resolved by SDS-PAGE presented the expected mass of 124 kDa (Figure 2).

**Table 1:** **mass calculations for DR_2577 hexamers, dimers and monomers defined by different methods**.

**Identification technique**	**Experimental mass (kDa)**	Oligomerization index^*^	**Oligomeric state**
SEC	>700 (Out of linearity)	–	–
BN-PAGE	769.4 (First band)	6.22	Hexamer
	736.7 (Second band)	5.95	Hexamer
SDS-PAGE (standard denaturation)	278 (First band)	2.25	Dimer
	268.2 (Second band)	2.17	Dimer
SDS-PAGE (harsh denaturation)	139.2	1.12	Monomer

*The calculations are based on the theoretical mass deducted from the primary sequence of DR_2577 (http://www.uniprot.org/uniprot/Q9RRB6)*.

* *Experimental mass/theoretical mass of the monomer*.

### DR_2577 monomers contain intermolecular disulfide bonds

SDS-PAGEs of samples not treated by a strong denaturation shows an exclusive presence of dimers, suggesting that DR_2577 might be prone to form specific intermolecular disulfide bonds. The DR_2577 sequence contains only two cysteine residues localized in the amino acid residues 896 and 929, respectively. In order to clarify the possible bases of the presence of dimers we have performed a basic analysis on the protein sequence, using the software DiANNA (Ferre and Clote, 2005a,b, 2006) for disulfide bonds prediction (http://clavius.bc.edu/~clotelab/DiANNA/), and found that only the residue 929 is likely to be a semi-cystine (and hence be prone to form a disulfide bond) so that only this residue may be potentially part of a disulfide bond (Supplementary Table 1). In the light of these facts it is most likely that two monomers could make a bridge by the interaction of the residues 929 from two different monomers entertaining an intermolecular disulfide bond.

### DR_2577 occurs in form of hexamers under native conditions

**Figure 3:**
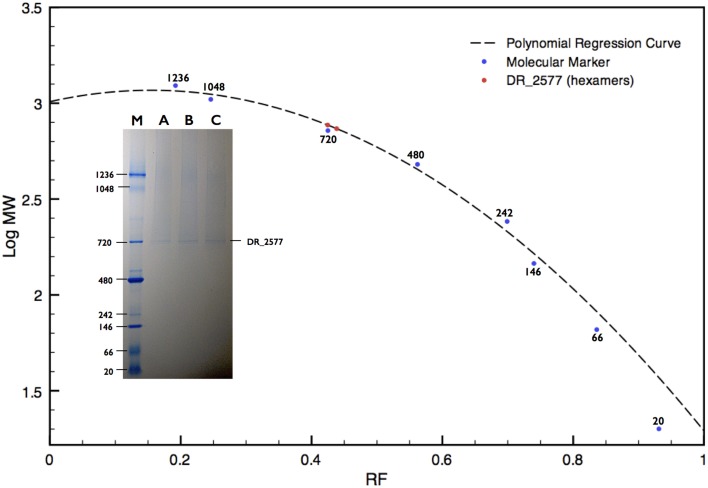
**BN-PAGE analysis of the DR_2577 sample eluted from the main SEC peak**. A polynomial regression curve is shown (dotted line) calculated on the base of the retardation factors of the molecular standard (blue dots). The two red dots on the polynomial regression curve show the calculated mass found for the DR_2577 complexes. The insert shows a typical DR_2577 sample (A, B, and C) separated by SEC and resolved by BN-PAGE; the lane M indicates the standard with the corresponding molecular weights.

DR_2577 showed small retention volumes when separated by SEC, strongly suggesting that under native conditions the protein could occur as a high molecular weight complex (Figure 1). With the aim to have a more detailed description of DR_2577 oligomeric state, we used the retention volumes of the SEC profiles and defined the size of the DR_2577 peak with respect to molecular standards. By this analysis a calculated mass out of the linearity range for this system was obtained (Figure 1; Table 1) not allowing to identify a precise size of the complex but showing that the complex has a mass greater than 700 kDa. Next, we performed a similar analysis by means of BN-PAGEs. Due to the typical auto-assembling properties of S-layer proteins (Pum et al., 2013), we performed these experiments loading about 0.1 μg of protein in order to reduce the tendency to smear that appears when the protein sample is loaded at concentrations greater than 0.5 μg. Using this method the sample was resolved into two isoforms (Figure 3) in which the heaviest band appeared as the most representative. The size of the complexes was estimated for both bands confirming that the protein occurs as a homo-oligomeric complex which can carry masses of ~740 and 770 kDa, respectively (Figure 3; Table 1). According to this finding and considering that a DR_2577 monomer accounts for 124 kDa it is reasonable to conclude that *in vivo* the DR_2577 complexes occur in form of hexamers (Table 1).

## Discussion

### DR_2577 has a characteristic hierarchical organization of its oligomeric states

Growing evidence suggests a primary role of the protein DR_2577 in the structural organization of the S-layer of *D. radiodurans* (Rothfuss et al., 2006; Farci et al., 2014). From early works it was observed how the knockout mutants for this protein typically lack of their S-layer integrity and their ability to resist to extreme conditions (Rothfuss et al., 2006). More recently was reported how the incidence of DR_2577 in the cell wall is far from being secondary (Farci et al., 2014).

In this work was found that DR_2577 can be isolated in purity by several steps starting from extracted S-layers in which DR_2577 is shown to be a dominant component (Supplementary Figure 1).

The protein DR_2577 can be separated into two different bands, either of dimers, when analyzed by SDS-PAGE (Figure 2), or of hexamers, when analyzed by BN-PAGE (Figure 3; Table 1). In both cases the presence of a double band can be explained by the characteristic tendency of S-layer proteins to assemble into ordered structures so that patterns similar to the observed in the PAGE experiments may be ascribed to rate-limited self-association phenomena in which the presence of two bands may be attributed to the strong imbalance between the dominant assembling reaction (expected for these kind of proteins) and its inverse disassembling (Shunong et al., 1991). Such unbalance would also explain the absence of an expected third band related to monomers in SDS-PAGE and to dimers in BN-PAGE. However, it cannot be excluded, even if less likely, that the two bands observed in both PAGE experiments could represent two different isoforms of the same protein. These observations suggest that *in vivo* the dimeric form should be the structural unit from which homo-oligomeric complexes of higher order are built, providing a structural cohesion to the S-layer. This hypothesis is further supported by the observation that the dimeric structural unit was found to be exceptionally resistant, so that only harsh denaturing conditions such as long boiling or strong treatment with tris-2-carboxy-ethylphosphine were able to induce formation of monomers. These results are also consistent with our preliminary bioinformatic analysis indicating that out of the two residues of cysteine present in the DR_2577 sequence, only one is a strong candidate to entertain a disulfide bond, allowing for the formation of DR_2577 homo-dimers between the same cysteine of two different monomers. Such stability and resistance to denaturation were previously observed also on the SlpA protein from *Thermus thermophilus*, a homolog of DR_2577, providing a further indication of similarity (Berenguer et al., 1988; Castón et al., 1988). Integration of these results with the observations emerged from SEC and BN-PAGE analyses provided also evidence that DR_2577 is organized into hexamers which accordingly with the description provided above must be constituted by triads of stable dimers. Previously, DR_2577 was shown to be exclusively associated to three complexes having masses of about 860, 916, and 1117 kDa, respectively (Farci et al., 2014). In the present work we have found that the purified DR_2577 assembles into slightly smaller complexes of about 750 kDa (Table 1), suggesting that DR_2577 hexamers *in vivo* may occur in association with other proteins. According to this hypothesis it is most likely that, as well as in the purified form, DR_2577 present “*in vivo*” the same oligomeric behavior, basing its assembling properties on a precise hierarchical organization for which stable dimers originate from disulfide bonds between two monomers and hexamers from non-covalent interaction between three stable dimers.

## Conclusions

**Figure 4:**
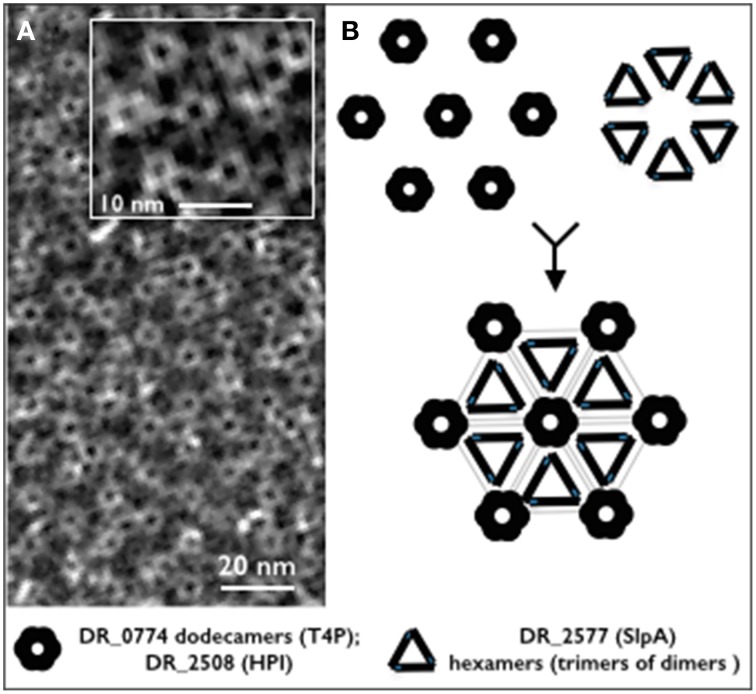
**(A)** Micrograph of a cell wall fragment showing the regular S-layer organization (from Farci et al., 2014). The inbox shows a magnified detail of the characteristic hexameric repetition for this S-layer; **(B)** schematic reconstruction of the hexameric unit on the base of the identified oligomeric profile showing a model with the possible localization of DR_2577 and DR_0774. The black and gray repetitions show trimers of DR_2577 dimers (hexamers), three hexamers are connected each other forming a closed structure which in its center host the DR_0774 channel and its related pore of HPI (DR_2508).

In spite of the deep detail into which the S-layer of *D. radiodurans* has been described, the proteome as well as the protein interactome for this structure are deeply unknown. In this work we have not only confirmed that DR_2577 is a major constituent of the S-layer, but also that its oligomeric states are peculiarly organized. In particular, we propose that DR_2577 monomers are assembled into stable dimers which are further combined in triads in order to constitute hexameric complexes. This elaborate organization, would constitute a structural unit of DR_2577 which could be associated in a regular fashion to the HPI assemblies contributing to the high level of cohesion characteristic for this S-layer. These conclusions are in agreement with the observation that oligomers of DR_2577 together with dodecameric channels of DR_0774 are structurally correlated with the hexagonally packed protein DR_2508 and represent altogether the essential components of this cell wall (Farci et al., 2014). We propose that the S-layer of *D. radiodurans* is composed by a regular organization of HPI assemblies, DR_0774 dodecameric channel and DR_2577 hexamers constituted by triad of dimers (Figure 4).

### Conflict of interest statement

The authors declare that the research was conducted in the absence of any commercial or financial relationships that could be construed as a potential conflict of interest.
