# Supplementary figures and images for: Purification and characterization of DR_2577 (SlpA) a major S-layer protein from Deinococcus radiodurans
Source: Front Microbiol. 2015 Jun 3;6:414. doi: 10.3389/fmicb.2015.00414 (PMC4419837; doi:10.3389/fmicb.2015.00414)

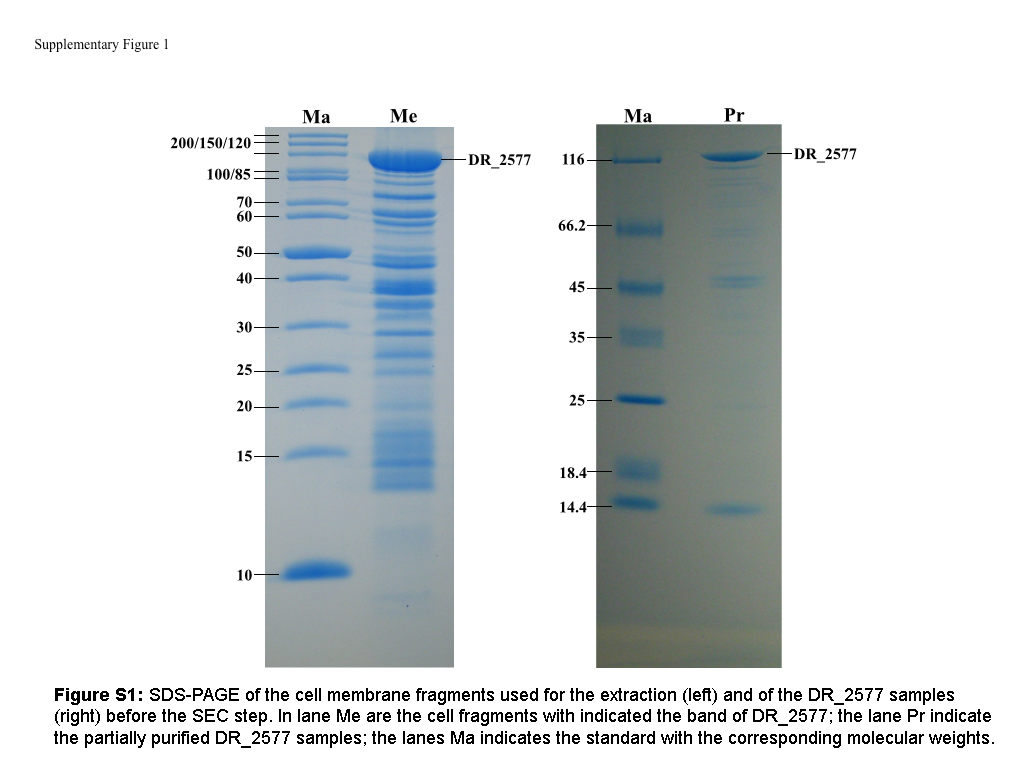

Supplement: Supplementary file 1 [file Image1.TIF]
