# Supplementary material for: Purification and characterization of DR_2577 (SlpA) a major S-layer protein from Deinococcus radiodurans
Source: Front Microbiol. 2015 Jun 3;6:414. doi: 10.3389/fmicb.2015.00414 (PMC4419837; doi:10.3389/fmicb.2015.00414)
Supplement: Supplementary file 2 [file Table1.PDF]

Supplementary Table 1

protein length (residues)	number of cysteine residues	position of cysteine residues	Half-cysteine	Free-cysteine	Ligand-bound	Disulfide Oxidation
**1186**	**2**	896	0.072511	**0.824304**	0.103185	0.0
		929	**0.499786**	0.410987	0.089227	**1.0**

Sup. table 1: disulphide bond prediction analysis using the software DiANNA (http://clavius.bc.edu/~clotelab/DiANNA/). The relevant parameters are shown with grey background. The cysteine residues may have scores between 0 and 1 indicating the degree of probability for a given state (half-cysteine, free cysteine and ligand-bound, disulphide oxidation).
